# Individual- and neighborhood-level factors influencing diet quality: a multilevel analysis using Korea National Health and Nutrition Examination Survey data, 2010-2019

**DOI:** 10.4178/epih.e2025043

**Published:** 2025-08-04

**Authors:** Dahyun Park, Min-Jeong Shin, S V Subramanian, Clara Yongjoo Park, Rockli Kim

**Affiliations:** 1 Interdisciplinary Program in Precision Public Health, Graduate School of Korea University, Seoul, Korea; 2 Multidisciplinary Research Center for Public Health in Complex System, Graduate School of Korea University, Seoul, Korea; 3 School of Biosystems and Biomedical Sciences, College of Health Science, Korea University, Seoul, Korea; 4 Harvard Center for Population and Development Studies, Cambridge, MA, USA; 5 Department of Social and Behavioral Sciences, Harvard T. H. Chan School of Public Health, Boston, MA, USA; 6 Department of Food and Nutrition, Chonnam National University, Gwangju, Korea; 7 Division of Health Policy and Management, Korea University College of Health Science, Seoul, Korea

**Keywords:** Diet, Neighborhood characteristics, Multilevel analysis, Social deprivation, Socioeconomic disparities in health

## Abstract

**OBJECTIVES:**

Although environmental factors influence lifestyle choices, few studies have examined how individual-level and neighborhood-level socio-demographic factors interact to affect diet quality in Korea. We investigated the associations between multilevel factors and diet quality among Korean adults and explored potential interactions by gender and age.

**METHODS:**

We conducted a cross-sectional analysis of 42,035 adults from 1,671 towns using data from the Korea National Health and Nutrition Examination Survey (2010-2019) and the Population and Housing Census of Korea (2010-2019). Individual-level variables included gender, age, education, income, number of household members, smoking, drinking, physical activity, and subjective health status. Neighborhood-level variables included residential area, housing type, number of restaurants per capita, population size, and the proportion of low-income households and older adults. Associations with the Korean Healthy Eating Index (KHEI) were assessed using 2-level hierarchical models.

**RESULTS:**

Of the total variance in KHEI, 5.2% was attributable to neighborhood-level differences. Individual-level factors explained 48.1% of variance at the neighborhood-level, while neighborhood-level factors accounted for an additional 12.4%. Individuals living in rural areas, non-apartment housing, neighborhoods with higher proportions of low-income households and older adults, or in areas with smaller populations, had lower KHEI scores than their counterparts. In random slope models with cross-level interaction terms, diet quality among adults aged 70 years and older varied significantly according to neighborhood- level characteristics.

**CONCLUSIONS:**

Both individual-level and neighborhood-level factors influence diet quality in Korea, with older adults being especially vulnerable to neighborhood characteristics. Multilevel approaches are needed to identify at-risk populations and improve dietary outcomes.

## GRAPHICAL ABSTRACT

[Fig f2-epih-47-e2025043]

## Key Message

Both individual- and neighborhood-level factors significantly influence diet quality in Korea. Rural residence, non-apartment housing, and neighborhoods with higher proportions of low-income or elderly residents were associated with lower Korean Healthy Eating Index (KHEI) scores. Older adults were especially vulnerable to adverse neighborhood environments, showing stronger cross-level interactions than younger adults.

## INTRODUCTION

Dietary quality refers to the overall healthfulness and nutritional adequacy of a diet, typically evaluated by its alignment with established dietary guidelines and its ability to provide essential nutrients while limiting harmful components. Suboptimal diet quality is a major risk factor for obesity [1] and non-communicable diseases (NCDs). Globally, 11 million deaths and 255 million disability-adjusted life years were attributable to dietary risk factors in 2017 [2]. To reduce the disease burden associated with NCDs, it is essential not only to quantify the health effects of dietary intake but also to investigate the diverse determinants of diet-related behaviors, especially in rapidly aging countries [3]. Prior research has established systematic differences in dietary patterns according to an individual’s socioeconomic status and neighborhood environment [4-6]. Socio-demographic characteristics such as gender, age, income, and educational attainment are associated with diet quality [7]. Individuals with higher socioeconomic status—through greater financial means, better physical access to healthy foods, and increased health knowledge—are more likely to consume whole grains, lean meats, low-fat dairy products, and fresh vegetables. In contrast, those with lower socioeconomic status tend to consume more fatty meats, refined grains, and added fats [8]. Neighborhood environments, including the availability and quality of grocery stores and restaurants, also influence dietary choices [9-13]. Moreover, the effect of neighborhood environments on food intake can differ depending on individual characteristics [14-16]; for example, adverse neighborhood exposures may disproportionately affect vulnerable or low-income individuals, leading to significant cross-level interactions. Therefore, it is necessary to investigate both the residential environment and individual characteristics from a multilevel perspective to understand their independent and combined associations with diet quality.

While the impact of neighborhood deprivation on health is well established [17,18], studies jointly assessing individual-level and neighborhood-level factors in relation to food choice or diet are limited [4]. Korea’s unique socio-demographic landscape—characterized by rapid economic development and social change in recent decades [19,20]—creates distinct multilevel determinants of diet quality that may differ from those in Western settings. Spatial analyses indicate that 73% of rural communities in Korea meet food desert criteria due to absolute retail shortages, while urban areas show localized insecurity clusters among younger populations [21,22]. The older population, which has the world’s longest life expectancy at 83.5 years [23] and a poverty rate of about 50% among older adults [24], faces additional barriers such as limited digital literacy and mobility challenges linked to geographic disparities in infrastructure [25]. As a result, susceptibility to the neighborhood food environment may vary by generation and could differ substantially from patterns observed in Western countries [4].

This study aimed to examine the associations between individual-level and neighborhood-level factors and diet quality among Korean adults using a multilevel modeling approach in a large, nationally representative sample. We also assessed whether these associations varied by gender and age, to identify vulnerable subgroups. We hypothesized that both individual and neighborhood characteristics would significantly influence dietary quality and that these effects would differ across demographic groups.

## MATERIALS AND METHODS

### Data source and subjects

This study adheres to the Strengthening the Reporting of Observational Studies in Epidemiology (STROBE) guidelines for cross-sectional studies. Data were obtained from the Korea National Health and Nutrition Examination Survey (KNHANES) for the years 2010 to 2019. Details of this nationally representative cross-sectional survey have been described elsewhere [26]. Participants were selected using a multistage, clustered probability design based on administrative units and housing type. The administrative units are structured in 2 layers: regions (larger units) and towns (smaller units). In this study, towns served as the main neighborhood unit. In the Korean administrative system, towns are defined as *dong*, *eup*, and *myeon*—the smallest units captured in KNHANES [26]. These units were chosen for their finer geographic resolution, better reflecting localized environmental contexts that may influence diet quality. For neighborhood-level variables, such as total population and the number of restaurants in each town, the KNHANES dataset was linked to demographic data from the Population and Housing Census of Korea (PHCK) [27] for the corresponding year.

Of the 70,115 KNHANES participants, 55,014 adults aged 19 years and older were eligible. Exclusion criteria included missing data on diet, education, income, alcohol consumption, smoking status, physical activity, subjective health, or neighborhood factors. Participants with implausible total energy intake (<500 or >5,000 kcal/day) were also excluded, following standard practices in nutritional epidemiology. The final analytic sample comprised 42,035 adults across 1,671 towns (Supplementary Material 1).

### Outcomes

Dietary quality was assessed using the Korean Healthy Eating Index (KHEI), developed to measure overall dietary quality based on Korean dietary guidelines [28]. The KHEI score was calculated from a single 24-hour dietary recall. KHEI components include 8 healthy dietary factors (breakfast intake; whole grains; total fruits; fresh fruits; total vegetables; fresh vegetables; protein-rich foods, including meat, fish, eggs, and legumes; and dairy products), 2 unhealthy dietary factors (sodium and energy from sweets and beverages), and 3 measures of energy intake (total energy, energy from carbohydrates, and energy from fat). Higher KHEI scores indicate more optimal diet quality (range, 5.87-89.72).

### Individual-level factors

At the individual-level, demographic characteristics, health behaviors, and subjective health status were examined. Age was categorized into 2 groups reflecting functional and social transitions in later life [29]: young adulthood (20-69 years) and older adulthood (≥70 years). Education was classified into 3 categories: middle school graduate or below, high school graduate, and college graduate or above. Household income was divided into quartiles. The number of household members was categorized as 1, 2, 3, or ≥4 persons. Health behaviors included self-reported frequency of alcohol consumption (never, <1/mo, 1-4 times/mo, ≥2 times/wk), smoking status (never, former, current smoker), and physical activity measured in metabolic equivalent of task (MET) hr/wk. Subjective health status was self-rated as “healthy,” “fair,” or “unhealthy.”

### Neighborhood-level factors

Neighborhood-level variables included residential area (urban or rural), housing type (apartment or non-apartment dwelling), number of restaurants per capita, total population of the town, proportion of residents with low household income, and proportion of elderly residents (≥70 years). Selection of these variables was guided by a social ecological framework and supported by empirical findings from Korea and international studies [30]. Rural and urban areas were defined according to administrative classifications from Statistics Korea and adopted in KNHANES [26]: areas designated as *dong* (neighborhood) were urban, while *eup* (town) and *myeon* (township) regions were rural, reflecting structural differences in population density, infrastructure, and access to services [31]. Housing type was included as a proxy for socioeconomic position and access to amenities such as grocery stores, green space, and health services. In Korea, apartments typically consist of vertically stacked units within planned residential complexes, offering convenient access to essential services, including grocery stores, retail shops, and playgrounds [32], and generally reflecting higher neighborhood resources. Non-apartment dwellings—such as detached houses, multiplex villas, and semi-basement units—are more varied in form and often associated with lower socioeconomic status and reduced access to amenities. Due to the KNHANES sampling design, all participants within the same town share the same housing type. Population size and restaurant density serve as indicators of commercial and food infrastructure, with these data sourced from the PHCK [27]. The proportions of low-income and elderly residents serve as indicators of structural vulnerability associated with dietary disparities [30]. These variables were selected to represent multiple contextual pathways influencing dietary behaviors. The proportion of residents with low household income and elderly residents was calculated as the number of KNHANES participants in the lowest income quartile or aged 70 years or older, respectively, divided by the total number of KNHANES participants in each town. All continuous neighborhood variables were categorized into quartiles, with higher quartiles indicating higher values.

### Statistical analysis

Participant characteristics at both the individual and neighborhood-levels are presented as mean±standard deviation (SD) for continuous variables and as percentages (counts) for categorical variables. Gender comparisons were conducted using the t-test for continuous variables and chi-square tests for categorical variables. Variation in diet quality and the effects of individual-level and neighborhood-level factors were assessed using sequential 2-level linear regression models. Model 1 was an empty (null) model that included only the random intercept for town and a fixed effect for survey year. Model 2 adjusted for individual-level variables, while model 3 further included neighborhood-level variables. Variable selection was based on prior literature and theoretical considerations as described above. For each model, the intra-class correlation coefficient (ICC) was calculated to estimate the proportion of variation in KHEI attributable to the neighborhood-level. Regression results are reported as coefficients with 95% confidence intervals (CIs). To assess whether the association between KHEI and neighborhood factors differed by gender and age, cross-level interaction terms were tested in model 4. As a sensitivity analysis, a 3-level regression model, including region as a third level above town, was performed to further assess variance partitioning in KHEI. All statistical analyses were conducted using Stata/SE version 13.0 (StataCorp. College Station, TX, USA). Statistical significance was set at p-value <0.05.

### Ethics statement

The study protocol was approved by the Institutional Review Board (IRB) of the KDCA (IRB No. 2018-01-03-PA). Informed consent was obtained as confirmed by the IRB.

## RESULTS

Forty-one percent of the participants were men, and the mean±SD age was 52.3±16.5 years (Table 1). One-third of participants lived alone. More than half (55.5%) resided in urban areas, and 21.9% lived in apartments. Women had higher mean KHEI scores than men (53.6±13.3 vs. 51.1±12.6, respectively, for total KHEI; p<0.001), with similar trends observed across most dietary components. The ‘year of survey’ variable indicates the year each participant was enrolled (2010-2019) and was included to account for potential temporal trends. The distribution of participants was relatively balanced across survey years, with no significant difference by gender (p=0.101).

In the baseline model (model 1), 5.2% of the variance in KHEI was attributable to the neighborhood-level (Table 2). After adjusting for individual-level factors (model 2), the ICC decreased to 4.1%, indicating that 48.1% of the neighborhood-level variation in KHEI was explained by individual-level characteristics. Most individual-level factors showed significant associations with KHEI. Women (β, 1.64; 95% CI, 1.32 to 1.96) was associated with better diet quality, whereas higher household income (β, -4.69; 95% CI, -5.08 to -4.31), higher education status (β, -4.23; 95% CI, -4.60 to -3.87), and better subjective health (β, -1.33; 95% CI, -1.66 to -1.00) were all associated with lower diet quality. When neighborhood-level factors were added in model 3, an additional 12.4% of the neighborhood-level variance was explained. Among these factors, living in rural areas (β, -0.39; 95% CI, -0.79 to 0.00), neighborhoods with a lower proportion of low household income (β, -0.58; 95% CI, -1.04 to -0.13), or a lower proportion of older adults (β, -1.46; 95% CI, -1.98 to -0.95) was associated with lower KHEI. Compared to residents of non-apartment housing, those living in apartments had higher KHEI (β, 1.20; 95% CI, 0.86 to 1.54). Population size and restaurants per capita were not significantly associated with KHEI.

**Figure 1.**
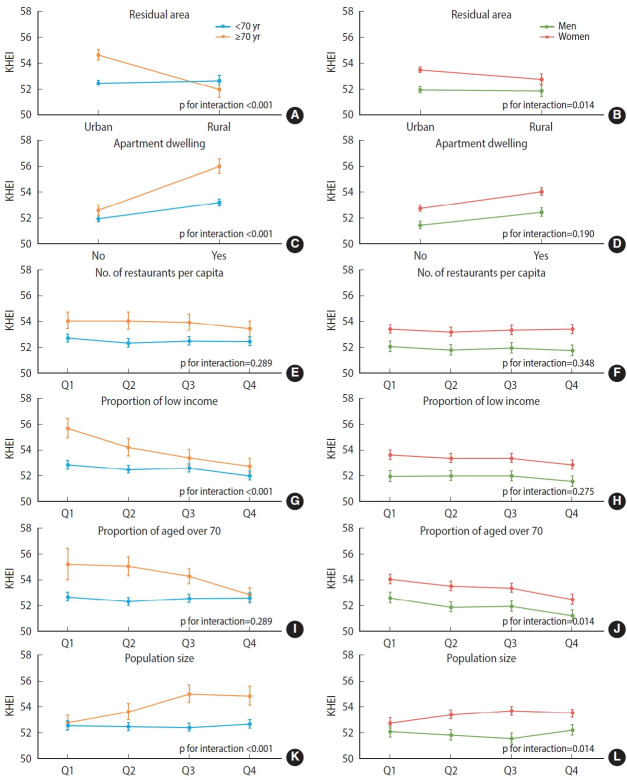
Random slope models of the Korean Healthy Eating Index (KHEI) showing cross-level interactions between neighborhood-level factors and demographic subgroups. (A–F) Age-stratified associations (<70 vs. ≥70 years) with residential area, apartment dwelling, number of restaurants per capita, proportion of low-income residents, proportion of residents aged ≥70 years, and population size. (G–J) Gender-stratified associations (men vs. women) with residential area, apartment dwelling, proportion of low-income residents, and population size. Interaction p-values are displayed within each panel. Interaction p-values indicate statistical significance, with significant differences observed for neighborhood of residence (p<0.05 for both age and gender groups), apartment residence (significant for age, p<0.001), percentage of low-income residents (significant for age, p<0.001), and population size (significant for all, p<0.05). KHEI scores were presented as means with error bars representing confidence intervals. These figures highlight the differential impact of neighborhood environment on dietary quality across demographic subgroups. Q, quartile; Q1-Q4 indicate lowest to highest quartiles.

In random slope analyses incorporating cross-level interaction terms (Figure 1), the association between certain neighborhood-level factors and KHEI varied significantly by age and gender. Among participants aged 70 years and older, those living in urban areas, residing in apartments, in neighborhoods with the lowest proportion of low household income, or in towns with larger populations, had significantly higher diet quality compared to their counterparts in other areas. In contrast, among participants younger than 70, differences in diet quality according to neighborhood-level factors were relatively minor. While women living in rural areas had slightly lower KHEI than those in urban areas, men’s diet quality was similar regardless of residential context. Given the limited gender differences, our interpretation focuses primarily on the more robust age-related interactions, which showed clearer and more consistent associations with neighborhood-level factors. As a sensitivity analysis, we conducted a 3-level linear regression model that included region as a third level. The results were consistent with those of the 2-level models, with only a modest proportion of variance attributable to the regional level (ICC=0.06).

## DISCUSSION

To the best of our knowledge, this is the first study in Korea to examine the influence of both individual-level and neighborhood-level factors on dietary quality and to assess their interactions with gender and age. This focus is especially important given Korea’s rapidly aging population and evolving food environment. Several notable findings emerged from our exploratory analysis, which can be summarized as 5 key points. First, approximately 5% of the variance in KHEI was attributable to town-level factors, underscoring the significant role of local environmental influences on dietary quality. Second, neighborhood-level characteristics—including urban residence, apartment dwelling, and higher population size—were positively associated with diet quality, whereas higher proportions of low-income residents were negatively associated. Third, neighborhood-level variables explained an additional 12.4% of the variance in dietary quality beyond what was accounted for by individual characteristics. Fourth, cross-level interactions revealed that older adults living in areas with a higher proportion of elderly residents had poorer diet quality, although, overall, older individuals tended to have better diets. Fifth, women’s diet quality was more negatively impacted by disadvantaged neighborhood conditions than men’s.

First, 5.23% of the total variance in KHEI was attributable to neighborhoods, highlighting neighborhoods as a key point of intervention for reducing disparities in dietary quality. Previous Korean studies have examined environments at broader geographic scales, such as the province or urban/rural level [33,34], which lack the fine-grained resolution considered here. Our sensitivity analysis, which simultaneously accounted for larger regions and smaller towns (Supplementary Material 2), found that regional-level factors explained only 0.3% of the variance in diet quality. This emphasizes that neighborhood-level features, including built and social environments within daily living areas, may be more closely associated with food choices. In Western contexts, studies have demonstrated that diet quality is shaped by differing policy environments and socio-cultural norms [30], such as wider availability of ultra-processed foods and more robust regulatory frameworks for nutrition labeling compared to many Asian countries [35].

Second, several neighborhood-level factors were significantly associated with dietary quality in Korea. Few studies have specifically evaluated local environmental impacts on food choice and diet quality [36]. In Korea, improvements in diet quality have paralleled economic development and policies that focus on individual decision-making, but disparities by education and income have widened over time [7]. While neighborhood-level factors included in this study have been linked to food choice or diet quality in other populations and in previous Korean research, they have not previously been assessed at the town level, which is the smallest administrative unit in Korea [18,34,37]. Our findings that urban residence, apartment dwelling, and larger population size are positively associated with diet quality suggest that higher population density may increase purchasing power, improve access to affordable healthy foods, and facilitate the dissemination of diet-related information, thus driving greater local demand for healthy foods [38].

Low household income was negatively associated with diet quality at both the individual and neighborhood-levels, consistent with previous reports from Korea [7] and Western populations [39,40]. Grocery stores and restaurants in poorer neighborhoods are less likely to offer fresh fruits, vegetables, or other healthy options [41]. Our findings align with NHANES data indicating that neighborhood socioeconomic status is positively associated with intake of, or home food availability for, fruits, dark green vegetables, and fat-free or low-fat milk [40,42]. The lack of association between restaurant density and diet quality in our study suggests that simple physical access to restaurants does not necessarily improve dietary quality. In the United States, higher restaurant density was linked to more frequent dining out and poorer diet quality among urban residents [43]. Some studies found that the types of restaurants or their proximity matter, though findings are inconsistent across populations [44,45]. Other analyses have considered food environments more broadly, including supermarkets and grocery stores, not just restaurants [46,47]. Further research on the types of restaurants and their influence on dietary quality in Korea is warranted, especially since Korea is a small, highly industrialized country with extensive public transportation and widespread internet connectivity, supporting reliable and rapid food delivery and minimizing the existence of food deserts.

Third, even after accounting for individual-level characteristics, neighborhood-level factors explained an additional 12.4% of the variance in dietary quality. This contribution is substantial, considering that the model already adjusted for a wide range of individual-level determinants known to influence diet quality in both Korean and other populations [37,38]. Our results thus provide a foundation for further research, highlighting that diet quality in Korea may be shaped not only by individual factors but also by the neighborhood environment within one’s immediate surroundings.

Fourth, significant cross-level interactions were observed between neighborhood-level factors (residential area, apartment dwelling, proportion of low-income residents, and population size) and individual age. Although diet quality was positively associated with individual age, paradoxically, living in neighborhoods with a higher proportion of older adults was associated with lower diet quality. This suggests that the effects of individual aging and of residing in older-adult-concentrated neighborhoods operate through distinct mechanisms. At the individual-level, higher KHEI scores among older adults may reflect increased health concerns, greater access to national nutrition policies (such as food assistance programs and free senior meals), and alignment with traditional Korean dietary patterns, which are generally optimal and more prevalent among older adults [24,26]. Conversely, the lower KHEI scores observed in neighborhoods with a higher proportion of older adults may result from reduced demand for fruits, vegetables, and meat among the elderly. Many of these foods have short shelf lives and are relatively expensive in Korea, leading to decreased availability and higher prices. In general, older individuals are more likely to be influenced by neighborhood characteristics due to retirement, decreased mobility, and limited use of e-commerce [43], while younger adults may be less affected by local food barriers given their greater ability to utilize e-commerce. Thus, older adults residing in areas with a high proportion of elderly residents may represent a priority population for public policy interventions aimed at improving diet quality. In random slope models with cross-level interactions, a significant interaction was also found between gender and neighborhood-level factors. While dietary quality is generally higher in women than in men [7], this study found that women are more susceptible to the negative influence of neighborhood-level factors, particularly living in rural or sparsely populated areas. This increased vulnerability may stem from women more frequently having the primary role in meal preparation, making them more sensitive to neighborhood characteristics [48]. Additionally, men may have higher rates of economic and social participation and are more likely to eat out, behaviors that are potentially less influenced by local neighborhood factors [49,50].

This study has several limitations. Most notably, the cross-sectional design and the use of single-day dietary assessments may not fully capture intra-individual variation in diet. Nonetheless, such assessments are generally sufficient for estimating group means in large populations. Although we included several key neighborhood-level indicators, other potentially important environmental factors—such as proximity to fresh food retailers, supermarket density, or walkability—were not incorporated [35]. Another limitation is that some neighborhood-level variables, such as the proportion of low-income households and elderly residents, were generated by interpolation, which may not precisely reflect actual neighborhood proportions. These limitations highlight constraints in publicly available spatial data at the town level in Korea [21]. Future research should incorporate more comprehensive neighborhood-level metrics, including geographic information system (GIS)-based measures of the retail and built environment, to further clarify contextual influences on diet quality. Because rural residence was significantly associated with lower diet quality, and several neighborhood variables also varied systematically by rural-urban classification [31], there is potential for multicollinearity and residual confounding. Although the sample size limited the possibility of stratified rural-urban analyses, future studies should pursue this approach to better elucidate how local context modifies the relationship between individual and neighborhood factors and diet quality. Despite these limitations, this study has important strengths. The use of a decade of national data and a large sample size provided strong statistical power. Unlike previous studies that focused on urban-rural differences or large administrative units such as provinces, this study assessed neighborhood environments at the smaller administrative town level, which may more closely influence individual dietary behaviors. While conducted in Korea, the findings may have broader relevance for other Asian countries undergoing rapid demographic transitions, such as population aging, urbanization, and widening socioeconomic disparities.

In conclusion, we found that neighborhood-level factors—including living in non-apartment housing, rural areas, or in neighborhoods with a high proportion of low-income or elderly residents—were associated with lower diet quality among Koreans. The elderly, in particular, appear more vulnerable to adverse neighborhood-level influences. These results suggest that public health strategies to improve diet quality should extend beyond individual-level interventions to incorporate spatially targeted approaches addressing neighborhood vulnerabilities. Future research should examine how built environment interventions and localized food policies might mitigate dietary inequalities shaped by residential context.

## Figures and Tables

**Figure f2-epih-47-e2025043:**
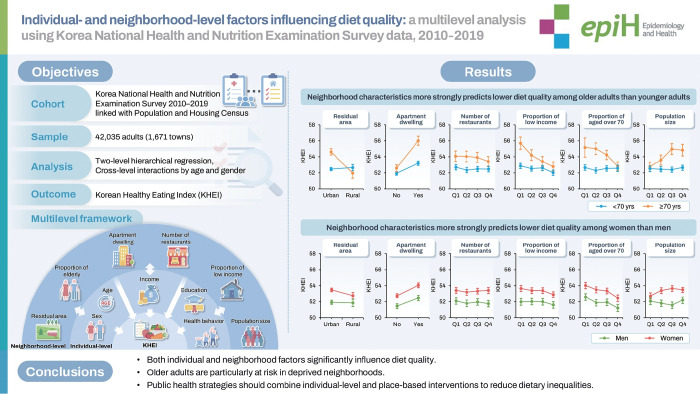


**Table 1.** Population characteristics of individual and neighborhood-level factors

Characteristics	Men (n=17,278)	Women (n=24,757)	Total (n=42,035)	p-value^1^
Outcome				
Korean Healthy Eating Index	51.1±12.6	53.6±13.3	52.6±13.1	<0.001
Eating breakfast	8.0±4.0	8.2±3.9	8.1±3.9	<0.001
Whole grain intake	2.1±2.3	2.2±2.3	2.2±2.3	0.132
Total fruit intake	1.9±2.1	2.7±2.2	2.4±2.2	<0.001
Fresh fruit intake	2.0±2.3	2.7±2.4	2.4±2.4	<0.001
Total vegetable intake	3.8±1.4	3.3±1.5	3.5±1.5	<0.001
Fresh vegetable intake	3.5±1.6	3.2±1.7	3.3±1.6	<0.001
Protein-rich food intake	7.1±3.1	6.6±3.3	6.8±3.2	<0.001
Dairy intake	1.8±3.5	2.2±3.8	2.0±3.7	<0.001
Sodium intake	5.2±3.6	7.2±3.2	6.3±3.5	<0.001
% Energy intake from sweets and beverages	7.1±4.3	7.2±4.3	7.2±4.3	0.468
% Energy intake from carbohydrates	2.4±2.1	2.1±2.1	2.2±2.1	0.656
% Energy intake from fat	3.2±2.2	3.0±2.2	3.1±2.2	<0.001
Total energy intake	3.2±2.2	3.1±2.2	3.1±2.2	0.875
Individual-level factors				
Age (yr)	52.5±16.6	52.1±16.4	52.3±16.5	0.015
Household income				<0.001
Highest quartile	27.9 (5,547)	25.7 (7,075)	26.6 (12,622)	
Upper middle quartile	26.8 (5,336)	25.3 (6,972)	26.0 (12,308)	
Lower middle quartile	25.6 (5,086)	25.9 (7,119)	25.7 (12,205)	
Lowest quartile	19.7 (3,916)	23.1 (6,359)	21.7 (10,275)	
Education				<0.001
College graduate and above	36.1 (6,306)	29.8 (7,468)	32.4 (13,774)	
High school graduate	33.9 (5,915)	29.4 (7,370)	31.3 (13,285)	
Middle school graduate and below	30.0 (5,240)	40.8 (10,212)	36.4 (15,452)	
No. of household members				<0.001
1	33.4 (6,684)	34.3 (9,488)	33.9 (16,172)	
2	24.8 (4,955)	24.4 (6,748)	24.5 (11,703)	
3	33.1 (6,623)	29.0 (8,013)	30.7 (14,636)	
≥4	8.7 (1,731)	12.4 (3,443)	10.9 (5,174)	
Smoking status				<0.001
Never	21.5 (3,866)	89.4 (22,920)	61.4 (26,786)	
Current smoker	35.5 (6,392)	4.9 (1,258)	17.5 (7,650)	
Former smoker	43.0 (7,735)	5.8 (1,474)	21.1 (9,209)	
Alcohol drinking				<0.001
Never	5.3 (953)	19.3 (4,961)	13.6 (5,914)	
<1/mo	24.8 (4,461)	42.5 (10,905)	35.2 (15,366)	
1-4 times/mo	34.0 (6,109)	28.3 (7,248)	30.6 (13,357)	
≥2 times/wk	36.0 (6,471)	9.9 (2,532)	20.6 (9,003)	
Physical activity (MET hr/wk)	1,454.1±2,542.8	1,054.1±2,142.9	1,218.3±2,323.8	<0.001
Self-rated health				<0.001
Healthy	33.6 (5,893)	26.7 (6,724)	29.5 (12,617)	
Fair	49.2 (8,636)	50.3 (12,665)	49.9 (21,301)	
Unhealthy	17.2 (3,012)	23.0 (5,787)	20.6 (8,799)	
Neighborhood-level factors				
Residential region				0.002
Urban areas	56.3 (11,257)	54.9 (15,203)	55.5 (26,460)	
Rural areas	43.7 (8,752)	45.1 (12,508)	44.6 (21,260)	
Apartment dwelling				<0.001
No	77.3 (15,459)	78.7 (21,803)	78.1 (37,262)	
Yes	22.7 (4,550)	21.3 (5,908)	21.9 (10,458)	
No. of restaurants per capita				0.004
Lowest quartile	24.6 (4,242)	25.6 (6,331)	25.2 (10,573)	
Lower middle quartile	24.7 (4,274)	25.3 (6,258)	25.1 (10,532)	
Upper middle quartile	24.9 (4,304)	24.7 (6,119)	24.8 (10,423)	
Highest quartile	25.8 (4,458)	24.4 (6,049)	25.0 (10,507)	
Population size				0.062
Lowest quartile	25.3 (4,371)	24.2 (5,979)	24.6 (10,350)	
Lower middle quartile	24.9 (4,300)	25.1 (6,222)	25.0 (10,522)	
Upper middle quartile	25.0 (4,323)	25.4 (6,295)	25.3 (10,618)	
Highest quartile	24.8 (4,284)	25.3 (6,261)	25.1 (10,545)	
Proportion of low household income				0.266
Lowest quartile	24.5 (4,232)	25.1 (6,220)	24.9 (10,452)	
Lower middle quartile	25.8 (4,458)	25.1 (6,213)	25.4 (10,671)	
Upper middle quartile	24.4 (4,213)	24.6 (6,095)	24.5 (10,308)	
Highest quartile	25.3 (4,375)	25.2 (6,229)	25.2 (10,604)	
Proportion of aged 70 or more				0.050
Lowest quartile	24.7 (4,270)	25.3 (6,250)	25.0 (10,520)	
Lower middle quartile	25.0 (4,310)	25.6 (6,344)	25.4 (10,654)	
Upper middle quartile	25.0 (4,323)	24.9 (6,160)	25.0 (10,483)	
Highest quartile	25.3 (4,375)	24.3 (6,003)	24.7 (10,378)	
Survey year				0.101
2010	7.7 (1,544)	7.7 (2,125)	7.7 (3,669)	
2011	8.9 (1,774)	9.2 (2,561)	9.1 (4,335)	
2012	7.9 (1,572)	8.5 (2,342)	8.2 (3,914)	
2013	10.7 (2,132)	10.9 (3,016)	10.8 (5,148)	
2014	10.1 (2,018)	10.4 (2,890)	10.3 (4,908)	
2015	10.7 (2,148)	10.5 (2,918)	10.6 (5,066)	
2016	10.4 (2,077)	10.7 (2,968)	10.6 (5,045)	
2017	11.4 (2,275)	10.6 (2,930)	10.9 (5,205)	
2018	10.9 (2,183)	10.7 (2,958)	10.8 (5,141)	
2019	11.4 (2,286)	10.8 (3,003)	11.1 (5,289)	

Values are presented as mean±standard deviation for continuous variables and percentage (count) for categorical variables. MET, metabolic equivalent of task.

1 From the t-test for continuous variables and the chi-square test for categorical variables.

**Table 2.** Associations between individual- and neighborhood-level factors and the Koreas Healthy Eating Index utilizing a multilevel regression model^1^

Variables	Model 1	p-value	Model 2	p-value	Model 3	p-value
Survey year						
2010	Reference		Reference		Reference	
2011	0.34 (-0.53, 1.22)	0.444	0.00 (-0.72, 0.72)	0.999	-0.17 (-0.86, 0.51)	0.619
2012	0.99 (0.09, 1.88)	0.030	0.43 (-0.31, 1.17)	0.254	0.49 (-0.22, 1.19)	0.175
2013	4.51 (3.67, 5.35)	<0.001	4.10 (3.41, 4.79)	<0.001	3.55 (2.89, 4.22)	<0.001
2014	5.37 (4.52, 6.22)	<0.001	4.83 (4.12, 5.53)	<0.001	4.41 (3.73, 5.09)	<0.001
2015	4.96 (4.12, 5.81)	<0.001	4.34 (3.64, 5.05)	<0.001	3.99 (3.31, 4.67)	<0.001
2016	4.90 (4.06, 5.74)	<0.001	4.41 (3.71, 5.11)	<0.001	3.99 (3.32, 4.67)	<0.001
2017	4.33 (3.49, 5.17)	<0.001	3.81 (3.11, 4.51)	<0.001	3.50 (2.82, 4.17)	<0.001
2018	4.32 (3.48, 5.16)	<0.001	3.70 (2.99, 4.40)	<0.001	3.31 (2.64, 3.99)	<0.001
2019	4.09 (3.25, 4.93)	<0.001	3.26 (2.56, 3.96)	<0.001	2.83 (2.15, 3.50)	<0.001
Individual-level factors						
Age (yr)	-		0.21 (0.20, 0.22)	<0.001	0.21 (0.20, 0.22)	<0.001
Gender						
Men	-		Reference		Reference	
Women	-		1.64 (1.32, 1.96)	<0.001	1.56 (1.24, 1.87)	<0.001
Household income						
Highest quartile	-		Reference		Reference	
Upper middle quartile	-		-0.96 (-1.27, -0.65)	<0.001	-0.78 (-1.09, -0.46)	<0.001
Lower middle quartile	-		-1.50 (-1.83, -1.18)	<0.001	-1.26 (-1.59, -0.93)	<0.001
Lowest quartile	-		-4.69 (-5.08, -4.31)	<0.001	-4.25 (-4.64, -3.86)	<0.001
Education						
College graduate and above	-		Reference		Reference	
High school graduate	-		-1.18 (-1.46, -0.89)	<0.001	-1.00 (-1.29, -0.71)	<0.001
Middle school graduate and below	-		-4.23 (-4.60, -3.87)	<0.001	-3.77 (-4.15, -3.40)	<0.001
No. of household members						
1	-		Reference		Reference	
2	-		0.59 (0.29, 0.89)	<0.001	0.66 (0.36, 0.95)	<0.001
3	-		1.03 (0.72, 1.34)	<0.001	1.25 (0.94, 1.56)	<0.001
≥4	-		-1.00 (-1.43, -0.58)	<0.001	-0.81 (-1.23, -0.38)	<0.001
Alcohol drinking						
Never	-		Reference		Reference	
<1/mo	-		0.93 (0.56, 1.29)	<0.001	0.86 (0.50, 1.23)	<0.001
1-4 times/mo	-		0.58 (0.19, 0.98)	0.004	0.49 (0.10, 0.89)	0.014
≥2 times/wk	-		-1.59 (-2.02, -1.16)	<0.001	-1.64 (-2.07, -1.20)	<0.001
Smoking status						
No	-		Reference		Reference	
Smoker	-		-3.92 (-4.30, -3.54)	<0.001	-3.89 (-4.27, -3.51)	<0.001
Former smoker	-		-1.27 (-1.63, -0.92)	<0.001	-1.30 (-1.65, -0.94)	<0.001
Physical activity (MET hr/wk)	-		0.36 (0.29, 0.44)	<0.001	0.36 (0.29, 0.44)	<0.001
Self-rated health						
Healthy	-		Reference		Reference	
Fair	-		-0.41 (-0.67, -0.15)	0.002	-0.39 (-0.65, -0.14)	0.003
Unhealthy	-		-1.33 (-1.66, -1.00)	<0.001	-1.31 (-1.64, -0.98)	<0.001
Neighborhood-level factors						
Residual area						
Urban areas	-		-		Reference	
Rural areas	-		-		-0.39 (-0.79, 0.00)	0.049
Apartment dwelling						
No	-		-		Reference	
Yes	-		-		1.20 (0.86, 1.54)	<0.001
No. of restaurants per capita						
Lowest quartile	-		-		Reference	
Lower middle quartile	-		-		-0.20 (-0.60, 0.20)	0.329
Upper middle quartile	-		-		-0.08 (-0.48, 0.33)	0.711
Highest quartile	-		-		-0.11 (-0.53, 0.30)	0.592
Population size						
Lowest quartile	-		-		Reference	
Lower middle quartile	-		-		0.35 (-0.08, 0.78)	0.109
Upper middle quartile	-		-		0.42 (-0.03, 0.86)	0.067
Highest quartile	-		-		0.58 (0.12, 1.04)	0.014
Proportion of low household income						
Lowest quartile	-		-		Reference	
Lower middle quartile	-		-		-0.15 (-0.56, 0.26)	0.475
Upper middle quartile	-		-		-0.15 (-0.58, 0.27)	0.485
Highest quartile	-		-		-0.58 (-1.04, -0.13)	0.011
Proportion of aged 70 or more						
Lowest quartile	-		-		Reference	
Lower middle quartile	-		-		-0.61 (-1.01, -0.20)	0.003
Upper middle quartile	-		-		-0.64 (-1.08, -0.21)	0.004
Highest quartile	-		-		-1.46 (-1.98, -0.95)	<0.001
Measure of variation or clustering						
Neighborhood-level variance (SE)	7.90 (0.48)		4.10 (0.34)		3.12 (0.29)	
Residual variance (SE)	141.31 (0.99)		129.03 (0.91)		128.89 (0.91)	
Intra-class correlation						
Neighborhood-level % (SE)	5.23 (0.31)		3.08 (2.48)		2.37 (2.20)	
Explained neighborhood-level variation (%)	-		48.1		12.4	

Values are presented as coefficient (95% confidence interval). MET, metabolic equivalent of task; SE, standard error.

1 Model 1: Null 2-level model adjusted for survey year fixed effects. Model 2: model 1+adjustment for individual-level factor variables. Model 3: model 2+adjustment for neighborhood-level factors.

## Supplementary materials

Supplementary Material 1. Associations between individual- and community-level factors and the Korea Healthy Eating Index using a three-level regression model

Supplementary Material 2. Participant flow chart
