# Supplementary material for: Individual- and neighborhood-level factors influencing diet quality: a multilevel analysis using Korea National Health and Nutrition Examination Survey data, 2010-2019
Source: Epidemiol Health. 2025 Aug 4;47:e2025043. doi: 10.4178/epih.e2025043 (PMC12673292; doi:10.4178/epih.e2025043)
Supplement: Supplementary Material 1. — Associations between individual- and community-level factors and the Korea Healthy Eating Index using a three-level regression model [file epih-47-e2025043-Supplementary-1.docx]

**Supplementary Material 1. Associations between individual- and community-level factors and the Korea Healthy Eating Index using a three-level regression model**

|  | **Model 1** | **Model 2** | **Model 3** |
| --- | --- | --- | --- |
|  | **Coef. (95%CI)** | **Coef. (95%CI)** | **Coef. (95%CI)** |
| **Individual-level factors** |  |  |  |
| Age, years |  | 0.21 (0.20, 0.22) | 0.21 (0.20, 0.22) |
| Gender |  |  |  |
| Men |  | Reference | Reference |
| Women |  | 1.61 (1.30, 1.93) | 1.55 (1.23, 1.86) |
| Household income |  |  |  |
| Highest quartile |  | Reference | Reference |
| Upper middle quartile |  | -0.94 (-1.25, -0.63) | -0.78 (-1.09, -0.46) |
| Lower middle quartile |  | -1.49 (-1.81, -1.17) | -1.25 (-1.58, -0.93) |
| Lowest quartile |  | -4.65 (-5.03, -4.26) | -4.24 (-4.64, -3.85) |
| Education |  |  |  |
| College graduate and above |  | Reference | Reference |
| High school graduate |  | -1.15 (-1.44, -0.86) | -0.99 (-1.27, -0.70) |
| Middle school graduate and below |  | -4.17 (-4.54, -3.80) | -3.75 (-4.13, -3.38) |
| Number of household members |  |  |  |
| 1 person |  | Reference | Reference |
| 2 people |  | 0.60 (0.30, 0.90) | 0.66 (0.36, 0.96) |
| 3 people |  | 1.07 (0.76, 1.38) | 1.26 (0.95, 1.57) |
| 4 people or more |  | -0.98 (-1.41, -0.55) | -0.80 (-1.23, -0.38) |
| Alcohol drinking |  |  |  |
| Never |  | Reference | Reference |
| Less than once a month |  | 0.88 (0.52, 1.25) | 0.84 (0.48, 1.21) |
| 1−4 times per month |  | 0.55 (0.15, 0.94) | 0.48 (0.08, 0.87) |
| 2 or more times a week |  | -1.62 (-2.05, -1.19) | -1.65 (-2.08, -1.22) |
| Smoking status |  |  |  |
| No |  | Reference | Reference |
| Smoker |  | -3.94 (-4.32, -3.56) | -3.90 (-4.28, -3.52) |
| Former smoker |  | -1.30 (-1.65, -0.94) | -1.31 (-1.66, -0.95) |
| Physical activity, MET hours per week |  | 0.37 (0.29, 0.44) | 0.36 (0.29, 0.44) |
| Self-rated health |  |  |  |
| Healthy |  | Reference | Reference |
| Fair |  | -0.39 (-0.65, -0.14) | -0.39 (-0.64, -0.13) |
| Unhealthy |  | -1.31 (-1.64, -0.97) | -1.30 (-1.63, -0.97) |
| **Neighborhood-level factors** |  |  |  |
| Residential area |  |  |  |
| Urban areas |  |  | Reference |
| Rural areas |  |  | -0.27 (-0.67, 0.13) |
| Apartment dwelling |  |  |  |
| No |  |  | Reference |
| Yes |  |  | 1.33 (0.98, 1.67) |
| Number of restaurants per capita |  |  |  |
| Lowest quartile |  |  | Reference |
| Lower middle quartile |  |  | -0.12 (-0.52, 0.28) |
| Upper middle quartile |  |  | 0.03 (-0.38, 0.44) |
| Highest quartile |  |  | 0.02 (-0.41, 0.44) |
| Population size |  |  |  |
| Lowest quartile |  |  | Reference |
| Lower middle quartile |  |  | 0.26 (-0.17, 0.70) |
| Upper middle quartile |  |  | 0.26 (-0.20, 0.71) |
| Highest quartile |  |  | 0.43 (-0.04, 0.89) |
| Proportion of low household income |  |  |  |
| Lowest quartile |  |  | Reference |
| Lower middle quartile |  |  | -0.10 (-0.51, 0.31) |
| Upper middle quartile |  |  | -0.07 (-0.50, 0.35) |
| Highest quartile |  |  | -0.47 (-0.93, -0.02) |
| Proportion of aged 70 or more |  |  |  |
| Lowest quartile |  |  | Reference |
| Lower middle quartile |  |  | -0.66 (-1.07, -0.26) |
| Upper middle quartile |  |  | -0.71 (-1.15, -0.28) |
| Highest quartile |  |  | -1.46 (-1.98, -0.94) |
| **Survey year** |  |  |  |
| 2010 | Reference | Reference | Reference |
| 2011 | 0.33 (-0.53, 1.19) | -0.02 (-0.73, 0.70) | -0.19 (-0.87, 0.49) |
| 2012 | 1.03 (0.15, 1.90) | 0.45 (-0.28, 1.17) | 0.47 (-0.23, 1.17) |
| 2013 | 4.44 (3.62, 5.27) | 4.04 (3.36, 4.73) | 3.54 (2.88, 4.20) |
| 2014 | 5.32 (4.48, 6.15) | 4.78 (4.08, 5.48) | 4.38 (3.70, 5.05) |
| 2015 | 4.91 (4.08, 5.74) | 4.29 (3.60, 4.99) | 3.96 (3.28, 4.63) |
| 2016 | 4.84 (4.02, 5.67) | 4.37 (3.68, 5.06) | 3.96 (3.29, 4.63) |
| 2017 | 4.29 (3.46, 5.12) | 3.77 (3.08, 4.47) | 3.46 (2.79, 4.13) |
| 2018 | 4.26 (3.44, 5.09) | 3.65 (2.96, 4.35) | 3.28 (2.61, 3.96) |
| 2019 | 4.06 (3.23, 4.88) | 3.23 (2.54, 3.92) | 2.79 (2.12, 3.46) |
| **Measure of variation or clustering** |  |  |  |
| 3-level variance: region (SE) | 0.37 (0.24) | 0.26 (0.13) | 0.12 (0.07) |
| 2-level variance: neighborhood (SE) | 7.45 (0.46) | 3.87 (0.33) | 3.06 (0.29) |
| Residual variance | 141.32 (0.99) | 129.00 (0.91) | 128.89 (0.91) |
| **Intraclass correlation** |  |  |  |
| Region level | 0.25 (0.12) | 0.19 (0.09) | 0.09 (0.05) |
| Neighborhood level | 5.25 (0.32) | 3.10 (0.26) | 2.37 (0.22) |

Model 1: Null two-level model adjusted for survey year fixed effects. Model 2: Model 1 + adjustment for individual-level factor variables. Model 3: Model 2 + adjustment for neighborhood-level factors. Coef., coefficient; 95%CI, 95% confidential interval; MET, Metabolic equivalent of task; SE, standard error.
