# Supplementary material for: Individual- and neighborhood-level factors influencing diet quality: a multilevel analysis using Korea National Health and Nutrition Examination Survey data, 2010-2019
Source: Epidemiol Health. 2025 Aug 4;47:e2025043. doi: 10.4178/epih.e2025043 (PMC12673292; doi:10.4178/epih.e2025043)
Supplement: Supplementary Material 2. — Participant flow chart [file epih-47-e2025043-Supplementary-2.docx]

**Supplementary Material 2. Participant flow chart**

| Total participants of KNHANES 2010-2019  ***n=70,115*** | |  |  |
| --- | --- | --- | --- |
|  |  |  | Excluded those  under the age of 19 |
|  |  |  | *(n=15,101)* |
| Adults aged 19 years and older  ***n=55,014*** | |  |  |
|  |  |  | Excluded those who had missing data   - Diet (n=6451) - Education (n=5281) - Income (n=195) - Frequency of alcohol consumption (n=184) - Smoking status (n=27) - Physical activity (n=66) - Subjective health (n=8) - Neighborhood factors (no town information or missing information on housing type; n=767) |
|  |  |  |  |
| final sample size for the analysis  n=42,035 (1,671 towns) | |  |  |
